# Severe Cerebral Salt Wasting Complicating Arginine Vasopressin Deficiency After Traumatic Brain Injury

**DOI:** 10.1210/jcemcr/luaf333

**Published:** 2026-02-04

**Authors:** Shi Hui Saw, Wayne S Cutfield, Craig A Jefferies, Benjamin B Albert

**Affiliations:** Starship Child Health, Te Whatu Ora—Health New Zealand, Te Toka Tumai Auckland, Auckland 1142, New Zealand; Liggins Institute, University of Auckland, Auckland 1023, New Zealand; Starship Child Health, Te Whatu Ora—Health New Zealand, Te Toka Tumai Auckland, Auckland 1142, New Zealand; Starship Child Health, Te Whatu Ora—Health New Zealand, Te Toka Tumai Auckland, Auckland 1142, New Zealand; Liggins Institute, University of Auckland, Auckland 1023, New Zealand

**Keywords:** arginine vasopressin deficiency, AVP-D, cerebral salt wasting, CSW, traumatic brain injury, TBI

## Abstract

Disturbances of water and sodium homeostasis may occur after traumatic brain injury. We report a 20-month-old girl who had a disturbance of water balance consisting of arginine vasopressin deficiency (AVP-D), extreme cerebral salt wasting (CSW), and finally permanent AVP-D following severe traumatic head injury. Magnetic resonance imaging of the brain showed transection of the pituitary stalk and the absence of a posterior pituitary bright spot. Her fluid balance disorder was also complicated by ACTH and TSH deficiency. The CSW phase was characterized by severe hyponatremia with dramatic polyuria and natriuresis and required aggressive fluid replacement with hypertonic saline in addition to vasopressin infusion and fludrocortisone. This case highlights the dynamic nature of fluid balance disorders after brain injury, the importance of recognizing the distinctive patterns of plasma and urine parameters in each condition, and the aggressive management required to treat severe cerebral salt wasting.

## Introduction

Approximately 1 in 4 patients with traumatic brain injury will have disturbances in water and sodium homeostasis in the first days following the incident [1]. These include the syndrome of inappropriate antidiuretic hormone secretion (SIADH), arginine vasopressin deficiency (AVP-D), and cerebral salt wasting (CSW), which pose significant challenges in diagnosis and management [1, 2]. Herein, we present the case of a child who had a disturbance of water balance consisting of AVP-D and extreme CSW, following severe traumatic brain injury.

## Case Presentation

A 20-month-old girl presented to the emergency department with severe trauma from a slow-moving car in a driveway. She sustained a crush injury to her head, with deformation of the left temporal region, and an impacted skull. A head computed tomography scan showed complex calvaria and base of skull fractures; malar and maxillary bone fractures; and subarachnoid, subdural, and intraventricular hemorrhage. She was intubated and sedated for a reduced level of consciousness.

## Diagnostic Assessment

On the first day after the trauma, she developed hypotension with polyuria, with a urine output of 13 mL/kg/hr. Investigations revealed peak plasma sodium of 162 mmol/L (reference range, 135-145 mmol/L), elevated plasma osmolality of 312 mmol/kg (reference range, 275-290 mmol/kg), and inappropriately low urine osmolality of 83 mmol/kg (reference range, 50-1200 mmol/kg) consistent with AVP-D. Urea was 34 mg/dL (5.6 mmol/L; reference range 10-32 mg/dL, 1.8-5.4 mmol/L), and creatinine was 0.28 mg/dL (25 µmol/L; reference range <0.45 mg/dL, <40 µmol/L) and subsequently was within the reference range for the remainder of the admission.

## Treatment

**Figure 1:**
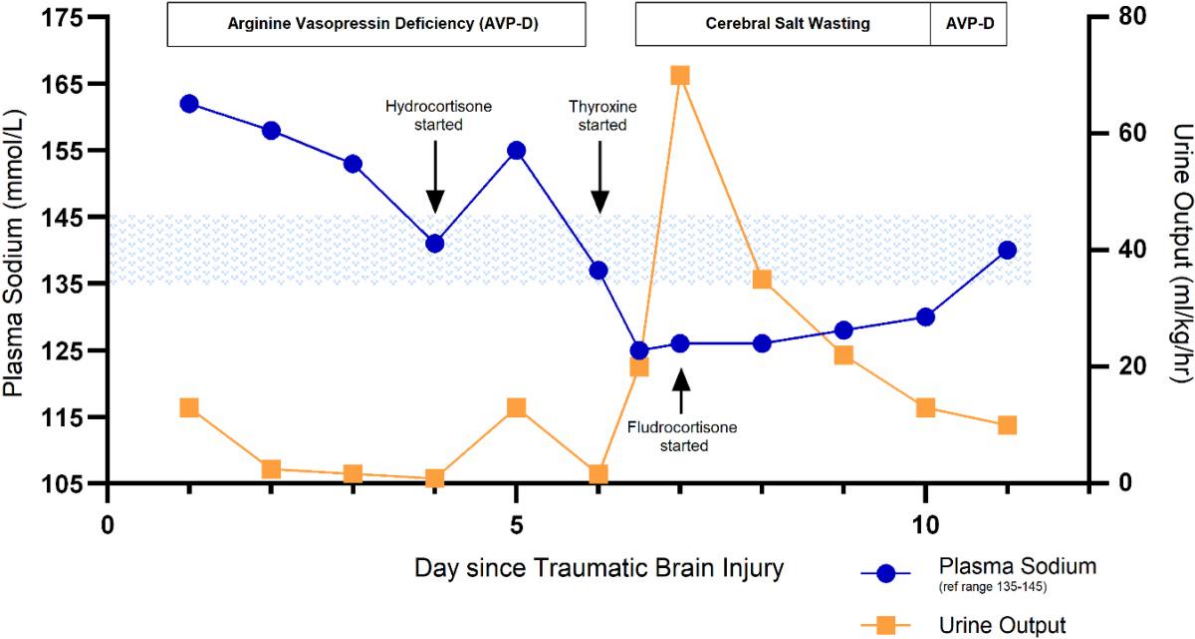
Plasma sodium concentration and urine output in the days following traumatic brain injury. The underlying water balance disorder is indicated.

She was treated with fluid replacement and aqueous vasopressin infusion (0.5 mU/kg/hr, titrated up to 2 mU/kg/hr). She responded to vasopressin, and urine output reduced to 2.4 mL/kg/hr, while plasma sodium levels gradually decreased to 153 mmol/L over 48 hours. Urine output and the plasma sodium across the 11 days of fluid instability are provided in Fig. 1.

On the third to fourth day posttrauma, her plasma sodium decreased rapidly from 155 to 140 mmol/L. A 3% saline bolus was given to prevent further decline in sodium, and the vasopressin infusion was tapered and subsequently stopped. Urine output reduced and remained low at 0.9 mL/kg/hr for 7 hours after stopping the vasopressin. The plasma osmolality was 287 mmol/kg, and urine osmolality was 695 mmol/kg. Her total fluid intake was restricted to 70% of estimated maintenance requirements. Her morning plasma cortisol was 2.3 μg/dL (56 nmol/L) (reference range, 6.1-18.2 μg/dL [170-500 nmol/L]), so she was started on IV hydrocortisone at 50 mg/m^2^/day for presumed ACTH insufficiency. Four hours after hydrocortisone treatment, AVP-D reemerged with a urine output of up to 14 mL/kg/hr and rising plasma sodium levels up to 155 mmol/L. Aqueous vasopressin infusion (0.5 mU/kg/hr) was restarted, and fluid losses were replaced with 0.45% saline. The decline in sodium level and reduction in urine output were assumed to be due to ACTH insufficiency, as adequate hydrocortisone replacement led to the AVP-D becoming evident again. While this event could also have been due to overtreatment with vasopressin, the urine output remained low for 7 hours after the vasopressin infusion was stopped, and aqueous vasopressin is reported to have a short half-life of approximately 24 minutes [3].

On the fifth day, her plasma sodium concentration reduced to 137 mmol/L, and her urine output reduced to 1.6 mL/kg/hr. Although these parameters are strictly normal, her fluid intake was limited to 70% maintenance, and aqueous vasopressin was stopped. Her sodium levels remained in the range of 137 to 140 mmol/L for 36 hours without AVP infusion, and her urine output was 2 to 3 mL/kg/hr, with a urine osmolality of 695 mmol/kg and a urine sodium concentration of 233 mmol/L (reference range: <20 mmol/L). However, thyroid function testing indicated TSH deficiency (free T4: 0.4 ng/dL [5.5 pmol/L], reference range 0.85-1.7 ng/dL [11-22 pmol/L]; free T3: 0.84 pg/mL [1.3 pmol/L], reference range 1.9-6.5 pg/mL [3-10 pmol/L]; TSH: 0.14 mIU/L, reference range 0.5-4.5 mIU/L), and she was started on levothyroxine 75 mcg daily. Hypothyroidism was thought to have contributed to the reduction in urine output and free water excretion. SIADH was also considered, although the patient did not fulfill the diagnostic criteria [Table 1].

**Table 1:** Diagnostic criteria of water balance disorders after traumatic brain injury [1, 4-6]

	Arginine vasopressin deficiency	Syndrome of inappropriate antidiuretic hormone	Cerebral salt wasting
Volume status	Hypovolemic	Euvolemic	Hypovolemic
Urine volume	>4 mL/kg/hr	Normal	>4 mL/kg/hr
Plasma sodium	>145 mmol/L	<135 mmol/L	<135 mmol/L
Plasma osmolality	>300 mmol/kg	<275 mmol/kg	<275 mmol/kg
Urine osmolality	<300 mmol/kg	>100 mmol/kg	>300 mmol/kg
Urine sodium		>30 mmol/L	>120 mmol/L

On the sixth day, she developed marked polyuria with a urine output of 10 to 20 mL/kg/hr, along with a rapid decline in plasma sodium concentration to a nadir of 125 mmol/L. She appeared delirious and dehydrated. Despite a low plasma sodium, urine sodium was markedly elevated at >250 mmol/L, and urine osmolality was 518 mmol/kg. N-terminal pro-B-type natriuretic peptide (NT-ProBNP) was elevated to 1065 pg/mL (126 pmol/L) (reference range, 296 pg/mL [<35 pmol/L]), supporting the diagnosis of CSW. She received aggressive replacement of urinary salt and water losses with 0.9% saline and multiple 3% saline boluses to maintain a sodium of 130 to 135 mmol/L. Fludrocortisone 0.2 mg twice daily was initiated, but the polyuria worsened, reaching a peak of 70 mL/kg/hr, and the hyponatremia persisted despite hypertonic saline boluses. Aqueous vasopressin infusion was restarted for possible concomitant AVP-D, and the dose was titrated up to 3 mU/kg/hr. Subsequently, the urine output decreased to 39 mL/kg/hr, suggesting a partial response. Additionally, a continuous 3% saline infusion was introduced and titrated up to 40 mL/hr.

From day 7 until day 10, the child had persistent hyponatremia (126-130 mmol/L) and high ongoing urinary sodium losses (urinary sodium concentration 200 mmol/L). Her urine output gradually decreased from 35 to 10 mL/kg/hr. On the 11th day posttrauma, her plasma sodium rose to 142 mmol/L, and urine sodium decreased to 50 mmol/L. She remained polyuric with a urine output of 10 mL/kg/hr. NT-ProBNP had reduced to 338 pg/mL (40 pmol/L) in keeping with AVP-D. Three percent saline infusion and fludrocortisone were stopped. She was started on IV desmopressin 1 mcg as needed, which was transitioned to regular oral desmopressin 50 mcg twice daily on day 18.

## Outcome and Follow-up

She was discharged home after 28 days, treated with oral desmopressin 50 mcg twice daily, levothyroxine 75 mcg daily, and hydrocortisone 2.5 mg twice daily. Her brain magnetic resonance imaging showed transection of the pituitary stalk and absence of the posterior pituitary bright spot, as well as rupture of the left optic nerve, which had led to blindness.

## Discussion

This case report illustrates a rare clinical finding of AVP-D, hypocortisolism, hypothyroidism, and CSW preceding and coexisting with each other, necessitating complex responsive management. The coexistence of AVP-D and CSW has been reported in severe central nervous system infection and after neurosurgery, hypoxic-ischemic events, head injury, and spontaneous intracranial hemorrhage [4, 7, 8]. The presence of transection of the pituitary stalk with severe diffuse brain injury including subarachnoid hemorrhage likely accounts for the complex time-course in this case.

AVP-D following traumatic brain injury in childhood, while well recognized, is uncommon; in a large study of 198 cases, no instances of AVP-D were reported [9]. Hyponatremia occurs in approximately 20% of survivors of traumatic brain injury. It can be caused by SIADH, CSW, excessive use of vasopressin replacement, ACTH/cortisol deficiency, or hypothyroidism [1, 4, 5].

Cortisol and thyroid hormones regulate renal blood flow and water excretion, so their deficiency can mask or appear to improve AVP-D. In this patient, pituitary stalk transection caused both deficiencies, and the identification of these deficiencies during a period of AVP-D coincided with reductions in urine output and plasma sodium. Urine output increased after replacement of the cortisol deficiency. Thyroid hormone deficiency was identified during a brief period, which could also have represented developing SIADH. SIADH was never proven because of treatment with 3% saline infusion to prevent hyponatremia. However, it is also possible that this event was solely due to thyroid hormone deficiency and not SIADH. The response to thyroid replacement could not be assessed due to the sudden onset of CSW.

There is controversy as to whether CSW is a disorder separate from SIADH, particularly when hyponatremia occurs in adults with subarachnoid hemorrhage [5, 6]. Elevated urine output and hypovolemia are the key determinants that differentiate CSW from SIADH, which were distinct features of our case [Table 1].

The pathophysiology of CSW is postulated to be due to abnormally elevated circulating natriuretic peptides, which inhibit the renin-angiotensin-aldosterone system and decrease renal sodium reabsorption. In the presented case, NT-proBNP was elevated during the CSW event but normalized when the CSW resolved. However, not all CSW cases show elevated BNP, and impaired sympathetic renal control may also play a role by lowering renin and aldosterone, reducing proximal sodium reabsorption, and dilating the afferent arteriole to increase glomerular filtration [5]. In a small case series, only 2/7 children with CSW had elevated BNP, while renin levels were suppressed or in the low-normal range, suggesting the importance of reduced sympathetic activity in the pathophysiology of CSW [10].

The diagnostic utility of NT-proBNP measurement is not clear, particularly in fluid balance disorders. The normal range in young children is wide (eg, age 1-3 years, 39-675 pg/mL), and while NT-proBNP may be may be increased in CSW, it is not consistently elevated [10, 11]. Data from an adult population indicate that in SIADH, mean NT-proBNP levels are increased, but there is significant overlap with euvolemic controls [12]. However, NT-proBNP is more useful in pediatric heart failure, as in combination with clinical scoring, a concentration greater than 598 pg/mL is diagnostic of heart failure [13]. The limited reliability of NT-proBNP in fluid balance disorders emphasizes that CSW should be diagnosed based on the combination of natriuresis and polyuria [14].

This patient had dramatic polyuria of up to 70 mL/kg/hr (normal range 1-2 mL/kg/hr) [15], which is close to the patient's estimated blood volume (75-80 mL/kg) each hour [16]. Aggressive fluid replacement was required, and any interruption risked life-threatening shock. The urinary sodium loss was also substantial, leading to persistent hyponatremia despite the replacement of urine output with normal saline. This occurred because the sodium concentration in the urine exceeded that of normal saline (154 mmol/L). Therefore, continuous 3% saline infusion was required in addition to the volume replacement, which was predominantly with 0.9% saline.

While aggressive replacement of urine sodium loss and water loss is the cornerstone of CSW management, fludrocortisone is a rational adjunct. Fludrocortisone is expected to augment plasma sodium concentration and intravascular volume by increasing sodium reabsorption in the renal proximal tubule [6, 17]. Vasopressin infusion was also started during CSW, when there was severe hyponatraemia and polyuria. AVP-D may have contributed to the severity of the polyuria, and the patient was expected to have remained AVP-deficient due to her brain pathology. Further, even if there had been functioning AVP secretion, it would be expected to be suppressed by the low serum osmolality [17].

Salt and fluid balance disorders after head injury can be severe and dynamic. Continual reassessment of fluid balance and plasma and urine sodium is required, as well as consideration of the potential development of pituitary hormone deficiencies.

## Learning Points

- Salt and fluid balance disorders after a head injury can be severe and dynamic.
- Fluid balance, sodium levels, and pituitary function must be continually reassessed to guide treatment.
- CSW can lead to extreme fluid and salt loss of life-threatening severity, and treatment must be proactive to replace these losses.

## Data Availability

Data sharing is not applicable to this article as no datasets were generated or analyzed during the current study.
